# Safety of Cerebrolysin for Neurorecovery after Acute Ischemic Stroke: A Systematic Review and Meta-Analysis of Twelve Randomized-Controlled Trials

**DOI:** 10.3390/ph14121297

**Published:** 2021-12-13

**Authors:** Stefan Strilciuc, László Vécsei, Dana Boering, Aleš Pražnikar, Oliver Kaut, Peter Riederer, Leontino Battistin

**Affiliations:** 1 Department of Neuroscience, Iuliu Hatieganu University of Medicine and Pharmacy, 400364 Cluj-Napoca, Romania; 2 Department of Neurology, University of Szeged, H-6725 Szeged, Hungary; vecsei.laszlo@medu-szeged.hu; 3 SRH Gesundheitszentrum Bad Wimpfen, 74206 Bad Wimpfen, Germany; ddboering@yahoo.de; 4 Institute for Neurological Sciences, Queen Elisabeth University, G51 4TF Glasgow, Ireland; ales.praznikar@gmail.com; 5 Department of Neurology, University of Bonn, Regina-Pacis-Weg 3, 53113 Bonn, Germany; Oliver.Kaut@ukbonn.de; 6 Clinic and Polyclinic for Psychiatry, Psychosomatics and Psychotherapy, University Hospital of Würzburg, 97080 Würzburg, Germany; peter.riederer@uni-wuerzburg.de; 7 Department of Psychiatry, University Southern Denmark, 5230 Odense, Denmark; 8 Department of Neurosciences, University of Padova, 35122 Padova, Italy; leontino.battistin@unipd.it

**Keywords:** ischemic stroke, safety, Cerebrolysin, neurorehabilitation

## Abstract

We performed a systematic search and meta-analysis of available literature to determine the safety profile of Cerebrolysin in acute ischemic stroke, filling existing safety information gaps and inconsistent results. We searched EMBASE, PubMed, and Cochrane Databases of Systematic Reviews and Clinical Trials up to the end of February 2021. Data collection and analysis were conducted using methods described in the Cochrane Handbook for Systematic Reviews of Interventions. All safety outcomes were analyzed based on risk ratios (RR) and their 95% confidence intervals. The meta-analysis pooled 2202 patients from twelve randomized clinical trials, registering non-statistically significant (*p* > 0.05) differences between Cerebrolysin and placebo throughout main and subgroup analyses. The lowest rate of Serious Adverse Events (SAE), as compared to placebo, was observed for the highest dose of Cerebrolysin (50 mL), highlighting a moderate reduction (RR = 0.6). We observed a tendency of superiority of Cerebrolysin regarding SAE in high dose treatment courses for moderate-severe ischemic stroke, suggesting some effect of the agent against adverse events. This comprehensive safety meta-analysis confirms the safety profile for patients treated with Cerebrolysin after acute ischemic stroke, as compared to placebo.

## 1. Introduction

Ischemic stroke continues to have overwhelming impact on health of populations and is expected to maintain its leading contribution to global mortality well into this century [1]. Studies have shown that post-stroke patients experience a wide range of adverse outcomes, such as aphasia, post-stroke anxiety, and depression, among others. Patient-level health outcomes for acute ischemic stroke have significantly improved in the last decade primarily because of superior overall case management, availability of tailored drug interventions, and advances in endovascular procedures. Nevertheless, health systems face a “care gap” particularly due to the ongoing COVID-19 pandemic, as well as other factors that hamper provision of quality services [2]. Several factors, including financing and infrastructure constraints, limited expertise, and clinical uncertainty, still prevent adherence to evidence-based clinical guidelines and optimal care pathways [3].

The concepts of neuroprotection and neurorecovery after stroke have been researched in many clinical settings in the past decades, with the aim of deciphering the specific biological interplay between various pharmacological interventions and the post-lesion endogenous defense mechanisms. Nevertheless, only a few trials in the last decades have succeeded in producing positive results in the broad field of brain protection and rehabilitation [4]. Numerous reasons could explain this outcome, such as unrobust methodological approaches that resulted in inconsistent evidence, therapeutic schemes that concentrated on suppressive strategies or the excessive research on interventions with single (monomodal) mechanisms of action.

Cerebrolysin is a combination of peptides that mimic the biological effect of neurotrophic factors, and amino acids obtained from highly purified lipid-free porcine brain proteins that promotes neurotrophic stimulation (survival and maintaining the phenotype of highly differentiated cells), neuroprotection against noxious agents, neuromodulation (e.g., changes in neuronal and synaptic plasticity), and metabolic regulation (i.e., against lactic acidosis and an increase in resilience against hypoxic conditions) [5]. Cerebrolysin has been shown to successfully pass the blood–brain barrier, despite various metabolic and biochemical processes that generally render targeting central nervous system recovery difficult from a pharmacological standpoint [6,7,8]. Randomized clinical trials have highlighted the efficacy and safety of the multimodal intervention for motor and neurological function recovery following AIS [9,10].

Cerebrolysin is recommended in several clinical practice guidelines as a pharmacological intervention for ischemic stroke, for both the acute phase and post-stroke rehabilitation [11,12,13]. Previous meta-analyses on Cerebrolysin safety profile provided inconsistent results. This applies especially to the two largest most recent meta-analyses: Bornstein et al. 2018, including 1879 patients from nine randomized-controlled trials (RCTs) [14], and the review of Ziganshina et al. 2020, including 1601 patients from seven RCTs [15]. Our meta-analysis aimed to explore the safety profile of Cerebrolysin, using a broad approach in identifying and appraising the available literature.

## 2. Materials and Methods

### 2.1. Study Selection and Information Sources

We used the PICO framework to establish the research question for the systematic review and meta-analysis (population—ischemic stroke, intervention—Cerebrolysin infusion, comparator—placebo or saline, outcome—safety parameters, to be explained in detail further on). The protocol for this review is available in the OSF registry, https://osf.io/cxufq accessed on 18 November 2021, [16]. Before starting the implementation of the project, we screened for similar reviews in the PROSPERO international prospective register of systematic reviews to avoid duplicating this effort.

### 2.2. Inclusion and Exclusion Criteria

We included randomized, double-blind, placebo-controlled, clinical studies completed until February 28th, 2021, and assessing the safety of Cerebrolysin as add-on treatment to standard care of ischemic stroke. Only articles published as full-text articles were considered as eligible for inclusion in this meta-analysis. No restrictions were placed on language, publication (year, type, or status), study endpoint (duration, length of follow-up, type of outcome measures) or treatment intervention (treatment window, dosage, frequency, or duration). If publications were not providing all details necessary for a comprehensive safety evaluation, supplementary study documents, such as study protocols, or clinical study reports, were requested from the original authors (grey literature).

Studies that did not provide outcome data or data usable for the meta-analysis as well as studies that did not meet the inclusion criteria were excluded. Safety parameters were adverse events, serious adverse events, non-fatal serious adverse events, and death, defined in compliance with current European Medicines Agency definitions described in the Note for guidance on clinical safety data management: definitions and standards (CPMP/ICH/377/95).

Information was sourced from Embase, PubMed and the Cochrane Database of Systematic Reviews up to end of February 2021. To further identify studies for this review, we also screened major review references and study registries (ClinicalTrials.gov, https://clinicaltrials.gov/; ISRCTN registry, http://www.isrctn.com/, accessed on 2 April 2021). We contacted authors of unpublished but registered studies and the producer of Cerebrolysin, to provide additional evidence and references for the meta-analysis. The search term “Cerebrolysin” was applied to all electronic database searches. The search strategy for Embase was (‘cerebrolysin’/exp OR Cerebrolysin) and for PubMed it was (“cerebrolysin” (Supplementary Concept) OR “cerebrolysin”(All Fields)). No filters were used. Article details were then exported and listed using a spreadsheet. Duplicate entries were removed automatically based on digital object identifiers and manually based on titles. Two independent reviewers (S.S. and D.B.) carried out the review, resolving diverging assessments by consensus. Abstrakr software was used to facilitate screening titles and abstracts (when available). Further screening was performed manually based on available full texts. We translated the full-text reports of studies published in languages other than English that were deemed eligible based on an English abstract. Studies from citation searches were screened for eligibility and cross-checked with already eligible entries.

Data from each included publication were extracted by the two reviewers working independently and using an extraction form that was devised for the study. Each included RCT was assessed for selection, performance, detection, attrition, and reporting bias, and other bias that might have been detected during the review process. Disagreement regarding the extracted elements, classification of evidence, or assessment of effect size was resolved by consensus; if consensus was not obtained, a third team member was involved (L.B.). Inclusion of any supplements for a specific trial was documented in the footnotes of the RoB table. In addition, individual patient data (IPD) were obtained for the following RCTs: Gharagozli et al. 2011, Heiss et al. 2012, Lang et al. 2012, Muresanu et al. 2016, and Guekht et al. 2015 [9,17,18,19,20]. Aggregate data from publication and individual patient data were cross validated. In case of discrepancies the original authors were contacted for clarification. All discrepancies could be resolved and were related to different underlying data sets (safety, ITT, FAS). For one trial no information on AE and SAE could be retrieved [17]. This study was excluded from the corresponding analyses.

### 2.3. Statistical Analysis

The safety outcomes were as follows: all-cause deaths, patients with at least one adverse event (AE), patients with at least one serious adverse event (SAE), and patients with at least one non-fatal serious adverse event (NFSAE). All safety outcomes were analyzed based on risk ratios (RR) and their 95% confidence intervals (CI). In one study no information was available on AE and SAE. This study was omitted from the corresponding analysis. We applied a random effects model (DerSimonian–Laird), based on the risk ratio (RR) as effect size for the binary safety criteria. Effect sizes were presented with 95% CIs and associated *p*-values. Heterogeneity was assessed by means of the I-squared (I2) procedure. All meta-analyses were performed using Revman (Version 5.4, The Cochrane Collaboration, London, England). In addition to the pooled analyses across all included randomized trials, sensitivity analyses were performed using the following stratification categories, including subsequent pooling across subgroups and formal tests for interaction:

- 20–30 mL vs. 50 mL
- 20–30 mL < 20 Days vs. 20–30 mL ≥ 20 Days
- 50 mL < 20 Days vs. 50 mL ≥ 20 Days
- Treatment Initiation Within 24 Hours of the stroke vs. Treatment Initiation > 24 Hours
- Studies published independently and available online.

For all subgroup analyses, tests for subgroup interaction and subgroup heterogeneity were performed based on Chi^2^ test and I^2^. A significance level of α = 0.05 was used a threshold for data interpretation. Risk of bias (RoB) assessment for the safety evaluations was performed using all available data from original publications. In unclear cases, supplementary information was requested from the original authors. Inclusion of any supplements for a specific trial was documented in the footnotes of the RoB table.

## 3. Results

**Figure 1:**
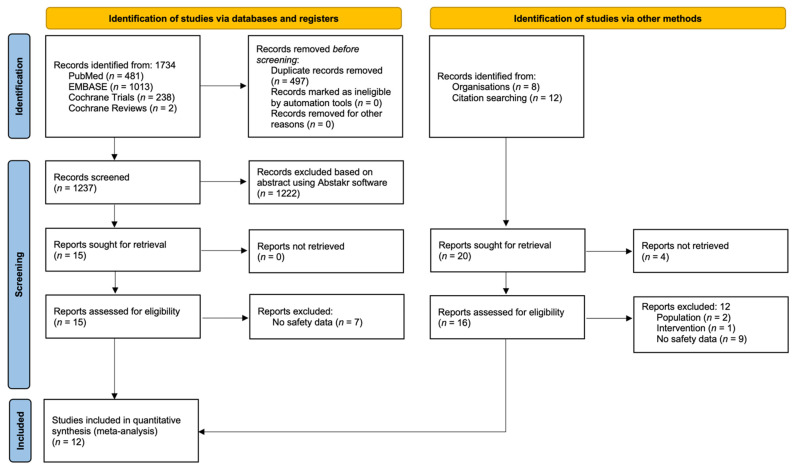
PRISMA flow diagram highlighting study selection process.

The systematic search process yielded 1734 results from databases and 20 entries via other methods described in the study methodology. A flow diagram of the search process is presented in Figure 1. Detailed risk of bias assessments is available in the Supplementary Material Table S1. Studies generally showed low risk of bias in the six analyzed domains (selection, performance, detection, attrition, reporting, and other), with the exception of three trials which had missing information, leading to unclear assessment results [17,21,22].

Twelve trials met the inclusion criteria, providing safety data for use of Cerebrolysin for 2202 from a total of 2274 randomized patients in the studies selected for formal analysis (Table 1).

All studies were declared as placebo-controlled, using saline solution. In some cases, special procedures were implemented to conceal the color of infusion lines. The baseline characteristics of studies are presented in Table 2.

### 3.1. Deaths

**Figure 2:**
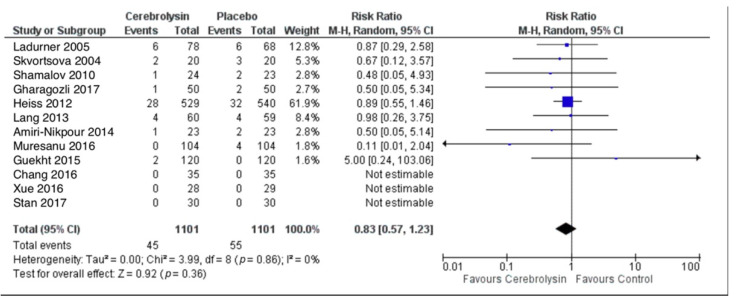
Deaths (All-cause); Comparison of Cerebrolysin versus Placebo, Safety Population, Random Effects, M-H, Risk Ratio (RR).

Crude pooling of deaths across studies resulted in a total of 45 deaths out of 1101 subjects treated with Cerebrolysin (4.1%), as compared to 55 deaths out of 1101 subjects treated with placebo (5.0%). Deaths were evaluated by means of the risk ratio (RR). The combined RR for deaths of all cause was resulting in a small superiority of Cerebrolysin with risk reduction in deaths by 17%, which was statistically not significant with *p* = 0.36 (RR = 0.83, 95%CI = 0.57 to 1.23, *p* = 0.36, random effects model, Figure 2).

### 3.2. Serious Adverse Events (SAE)

**Figure 3:**
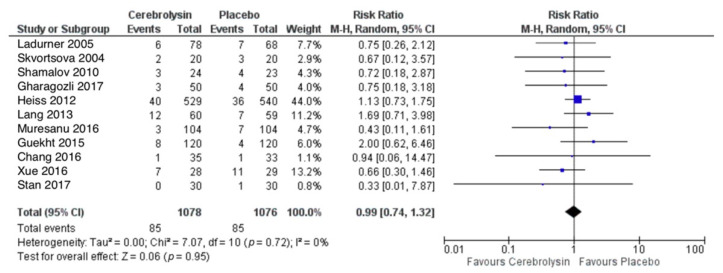
Serious adverse events (patients with at least one SAE); Comparison of Cerebrolysin versus Placebo in the Safety Population, Random Effects, M-H, Risk Ratio (RR).

SAE were reported in a total of 85 out of 1078 subjects treated with Cerebrolysin (7.9%), as compared to 85 out of 1076 subjects treated with placebo (7.9%). The combined RR for patients with at least one SAE showed no difference between the groups (RR = 0.99, 95%CI = 0.74 to 1.32, *p* = 0.95, random effects model, Figure 3).

### 3.3. Adverse Events (AE)

**Figure 4:**
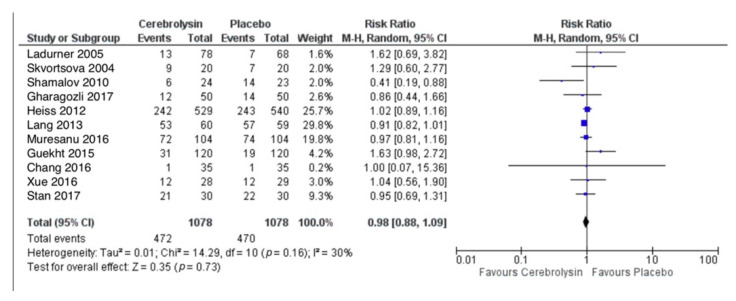
Adverse events (patients with at least one AE); Comparison of Cerebrolysin versus Placebo in the Safety Population, Random Effects, M-H, Risk Ratio (RR).

AE were reported in a total of 472 out of 1078 subjects treated with Cerebrolysin (43.8%), as compared to 470 out of 1078 subjects treated with placebo (43.6%). The combined RR for patients with at least one AE showed no difference between the groups (RR = 0.98, 95%CI = 0.88 to 1.09, *p* = 0.73, random effects model, Figure 4).

### 3.4. Non-Fatal Serious Adverse Events (NF-SAE)

**Figure 5:**
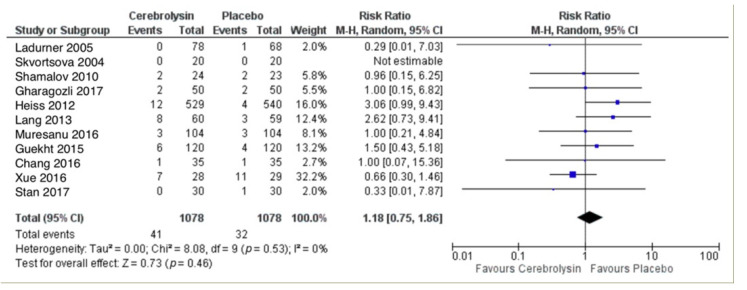
Non-fatal serious adverse events (patients with at least one NF-SAE); Comparison of Cerebrolysin versus Placebo in the Safety Population, Random Effects, M-H, Risk Ratio (RR).

NF-SAE were reported in a total of 41 out of 1078 subjects treated with Cerebrolysin (3.8%), as compared to 32 out of 1078 subjects treated with placebo (3.0%). The combined RR for patients with at least one NF-SAE showed a slightly higher rate in the Cerebrolysin group, which was statistically not significant with *p* = 0.46 (RR = 1.18, 95%CI = 0.75 to 1.86, *p* = 0.46, random effects model, Figure 5).

### 3.5. Sensitivity Analyses

All single subgroup results, as well as all formally combined subgroup results, were statistically not significant, well supporting the results of the crude pooling of all included randomized trials. Results from these analyses are present in Table 3. Effects for the 50 mL subgroup treated for 20 days or more could not be estimated based on identified data.

## 4. Discussion

This study aimed to systematically assess safety outcomes for patients who received Cerebrolysin for ischemic stroke in randomized, double-blind, placebo-controlled, clinical studies. Previous literature on this topic followed methodologically different pathways leading to diverging conclusions [14,15]. The safety of the neurotrophic factor-like drug has been previously evaluated in various studies, presenting heterogeneous results regarding demographics, time of inclusion, and administered dose, as well as time of follow-up.

To resolve the reported discrepancies between studies evaluating the safety of Cerebrolysin after acute ischemic stroke, the present meta-analysis aimed to include a maximum number of RCTs and patients, and to fill existing safety information gaps by following-up with primary source references and requesting additional material from original authors and the producer of Cerebrolysin. Aiming to provide additional clarity regarding the safety of the intervention is essential given its widespread use and recommendation in clinical guidelines [11,12], in conjunction with the immense global burden of ischemic stroke [1]. As the prevalence of risk factors that have been linked to this affliction (e.g., ageing, and lifestyle) are also increasing, future efforts to improve the armamentarium of interventions that aim to mitigate its effects must be mirrored by efforts for prevention [28].

Our pooled analysis of 2202 patients highlighted no indication for safety issues of Cerebrolysin. This was consistently observed throughout the pooled analyses of 12 RCTs, as well as throughout all subgroup analyses (*p*-values > 0.05). The least SAE rates as compared to placebo were found for the highest Cerebrolysin dose (50 mL), showing a moderate reduction in SAE as compared to placebo. In addition, there was a tendency for overall reduction in all-cause deaths. It is interesting to note that the least SAE and non-fatal SAE rates were found for the highest Cerebrolysin dose with > 25% risk reduction as compared to placebo.

The causes of SAE may be split into deaths and others, but these events are both SAE. Ziganshina et al. 2020 evaluated six studies for all-cause death (RR 0.9) [15]. However, for SAE and non-fatal SAE, they included only four studies. For the fatal SAE the above cited study included only three trials, even though information for fatal SAE was available in a total of six trials, and SAE was available for four studies. Gharagozli 2017 was evaluated for non-fatal SAE but not for fatal SAE (despite having one Cerebrolysin death and two placebo deaths) [17]. The reason for this approach may lie in the PICO of the review, namely, that “all of the deaths occurred within the seven-day acute-phase post-stroke period, owing to the severity of stroke”. Gharagozli et al. writes in the article, “three patients died in the acute phase due to stroke severity”. For consistency, exclusion of such patients from fatal SAE analysis, usually warrants a similar approach for non-fatal SAE. We therefore assert that the Ziganshina 2020 et al. fatal vs. non-fatal SAE evaluation faces two key limitations that are not formally addressed in the review: (1) reduction in fatal SAE analysis to studies with non-fatal SAE information only; and (2) the exclusion of one trial from fatal SAE analysis without specifying a general rule, so as to describe the selection as a special subset from the total fatal SAE population.

In our safety meta-analysis, we include 12 studies providing details on SAE. For all studies, fatal, and non-fatal SAE are explicitly reported as such in primary sources. Some trials had only few deaths but no other SAE (non-fatal = 0). One trial had no information on SAE [24]. The definition of SAE is not related to the presumed cause of the adverse event (e.g., “prolongation of existing hospitalization” is acknowledged as SAE regardless of causal relationship with the underlying disease). Additionally, the time of occurrence plays no role in SAE classification, except in cases where the event occurred within the timespan of the human drug trial. As part of the limiting factors of this meta-analysis, there was a large heterogeneity of the trials with respect to baseline stroke severity: NIHSS trial medians were reaching from 7 to 14. A stratified analysis on studies with mild (NIHSS < 8) versus moderate–severe (NIHSS ≥ 8) stroke provided no indication for impact on safety results (all interaction *p* ≥ 0.8), with one exception: for mild vs. moderate–severe stroke the test for subgroup differences regarding patients with at least one AE indicated moderate heterogeneity (I2 = 63.6%, *p* = 0.10), with lower risk ratios favoring Cerebrolysin in the moderate–severe subgroup (RR 0.95, *p* = 0.33), as compared to higher risk ratios in the mild subgroup (RR 1.26, *p* = 0.16). Another limitation is the restricted information on study conduct from some of the included trials despite special requests for provision of additional information, as well as absence of more prolonged longitudinal safety observations (6 months, 1 year), which were not available from randomized clinical trials. These should be considered within the framework of future study designs.

The main strength of the current paper is the inclusion of the largest number of studies on Cerebrolysin after stroke so far, comprising a total of 12 randomized double-blind trials. An important advantage is the inclusion of additional material, requested from the original authors if publications with summarized safety sections were not providing enough data for all safety outcomes of interest, a problem many such studies are confronted with. Therefore, a maximum of safety-related data could be obtained. Another strength is the homogeneity of the safety results across all sensitivity analyses, supporting the main result and demonstrating the robustness of the safety results across all analysis pathways.

This comprehensive safety meta-analysis shows a very good safety profile for patients treated with Cerebrolysin after acute ischemic stroke as compared to placebo. While none of the analyses provided evidence for safety issues, there was a tendency to superiority of Cerebrolysin regarding serious adverse events in high dose treatments and in moderate–severe stroke. Further randomized clinical trials are welcome to provide additional evidence based on longer follow-up duration and mixed or repetitive treatment cycles. Moreover, the development of effectiveness studies would also contribute to enhancing the strength of current assertions regarding the safety of this intervention.

## Figures and Tables

**Table 1:** Description of studies and populations included in formal analyses.

First Author and Year	Sample ^3^	Cerebrolysin Regimen		Comparator	Initiation Window	Endpoint	Countries	Baseline NIHSS
Ladurner, 2005 [21]	*n* = 146	50 mL/day for 121 days		Placebo (0.9% saline)	Within 24 h	CNS at day 21	Austria, Czech Republic, Hungary	CNS ^1^ 6.9 ^1^ 6.7 ^1^ NIHSS 9.2 ^5^ 9.6 ^5^
Skvortsova, 2004 [22]	*n* = 60	10 or 50 mL/day for 10 days		Placebo (0.9% saline)	Within 12 h	MRI infarct volume at day 30	Russia, Romania	13.1 ^1,4^ 12.6 ^1^
		+100 mg ASA/day for 10 days						
		+250 mg ASA/day for 90 days +pentoxifylline (days 1–21: 300 mg, days 22–90: 800 mg/day)						
Shamalov, 2010 [23]	*n* = 47	50 mL/day for 10 days		Placebo (0.9% saline)	Within 12 h	MRI infarct volume at day 30	Russia	7.7 ^1^ 8.6 ^1^
		+100 mg ASA/day for 10 days						
Gharagozli, 2017 [17]	*n* = 100	Day 1–7: 30 mL/day Week 2–4: 10 mL/day, 5 days/week		Placebo (0.9% saline)	Within 18 h	NIHSS at day 30	Iran	9.1 ^1^ 11.1 ^1^
		+ basic therapy						
Heiss, 2012 [18]	*n* = 1070	Cerebrolysin 30 mL/day for 10 days		Placebo (0.9% saline)	Within 12 h	Composite of NIHSS, mRS, BI at day 90	China, Hong Kong, South Korea, Myanmar	9 ^2^ 9 ^2^
		+100 mg ASA/day for 90 days						
Lang, 2013 [19]	*n* = 119	Cerebrolysin 30 mL/day for 10 days		Placebo (0.9% saline)	Immediately after rt-PA infusion	mRS at day 90	Austria, Croatia, Czech Republic, Slovakia, Slovenia	12.3 ^1^ 11.0 ^1^
		+rt-PA over 60 min			Within 3 h			
Amiri-Nikpour, 2014 [24]	*n* = 46	Cerebrolysin 30 mL/day for 10 days		Placebo	Within 6–24 h	NIHSS at day 30, 60, 90	Iran	14 ^2^ 14 ^2^
		+100 mg ASA						
Muresanu, 2016 [9]	*n* = 208	Cerebrolysin 30 mL/day for 21 days		Placebo	Within 24–72 h	ARAT at day 90	Romania, Ukraine, Poland	9.1 ^1^ 9.2 ^1^
		+ basic therapy						
Guekht, 2015 [20]	*n* = 240	Cerebrolysin 30 mL/day for 21 days		Placebo	Within 24–72 h	ARAT at day 90	Russia	7.5 ^1^ 6.8 ^1^
Chang, 2016 [25]	*n* = 70	30 mL/day for 21 days		Placebo (0.9% saline)	Within 7 days	FMA-T at day 29	Korea	8.4 ^1^ 7.0 ^1^
Xue, 2016 [26]	*n* = 84	Cerebrolysin 30 mL/day for 10 days		Placebo	Within 12 h	NIHSS and BI Day 30	China	13.3 ^1^ 12.7 ^1^
				NBP				
		+basic therapy						
Stan, 2017 [10]	*n* = 84	Cerebrolysin 30 mL/day for 10 days	Placebo		Within 48 h	NIHSS at Day 30	Romania	8.9 ^1^ 7.8 ^1^

^1^ Means (Cerebrolysin vs. placebo), ^2^ medians (Cerebrolysin vs. placebo), ^3^ all randomized groups, ^4^ 50 mL group ^5^ No NIHSS available, NIHSS derived from CNS using validated a conversion model [27].

**Table 2:** Demographic characteristics of studies included in the analysis.

Variables	Age (Mean; SD)		Male Gender (*n*; %)	
Study	Cerebrolysin	Placebo	Cerebrolysin	Placebo
Ladurner, 2005 [1]	65; 1.17	65; 1.32	47; 60.3	38; 55.9
Skvortsova, 2004 [2]	ages 45–85		n/a	
Shamalov, 2010 [3]	ages 45–85		n/a	
Gharagozli, 2017 [4]	69.0; 10.7	66.5; 12.2	27; 54%	26; 52%
Heiss, 2012 [5]	65.0; 12.22	65.6; 11.71	314; 59.6%	326; 60.4%
Lang, 2013 [6]	65.6; 11.30	67.0; 10.56	40; 66.7%	37; 62.7%
Amiri-Nikpour, 2014 [7]	60; 9.6	60.1; 10	12; 51.2%	10; 47.6%
Muresanu, 2016 [8]	64.9; 9.8	63.0; 10.6	70; 67.3%	63; 60.6%
Guekht, 2015 [9]	63.8		59.7%	
Chang, 2016 [10]	64.7; 10.1	63.0; 10.6	29; 82.9%	24; 72.7%
Xue, 2016 [11]	66.5; 8.1	68.4; 4.2	9; 45%	10; 50%
Stan, 2017 [12]	62.96; 10.9	65.23; 11.1	19; 63.3%	20; 66.5%

**Table 3:** Results of subgroup sensitivity analyses. Effect estimates risk ratios are computed using the Mantel–Haenszel method (M-H, random, 95% Confidence Interval).

Sample/Indicator	All Studies	Cerebrolysin Dose: 20–30 mL			Cerebrolysin Dose: 50 mL		Initiation		Studies Available Online
		All	<20 Days	>=20 Days	All	<20 Days	<= 24 h	>24 h	
Deaths									
No. studies	12	9	5	3	3	3	8	4	11
Sample size	2202	1969	1351	518	233	233	1624	578	1962
Effect estimate	0.83 (0.57, 1.23)	0.86 (0.55, 1.33)	0.88 (0.56, 1.39)	0.73 (0.02, 30.67)	0.75 (0.32, 1.76)	0.75 (0.32, 1.76)	0.84 (0.57, 1.25)	0.73 (0.02, 30.67)	0.81 (0.55, 1.20)
SAE									
No. studies	11	8	4	3	3	3	7	4	10
Sample size	2154	1923	1305	518	233	233	1578	578	1914
Effect estimate	0.99 (0.74, 1.32)	1.05 (0.77, 1.43)	1.07 (0.75, 1.54)	0.98 (0.34, 2.87)	0.72 (0.34, 1.52)	0.72 (0.34, 1.52)	1.00 (0.73, 1.36)	0.92 (0.38, 2.23)	0.95 (0.70, 1.28)
AE									
No. studies	11	8	4	3	3	3	7	4	10
Sample size	2156	1923	1305	518	233	233	1578	578	1916
Effect estimate	0.98 (0.88, 1.09)	0.97 (0.89, 1.05)	0.95 (0.88, 1.03)	1.18 (0.74, 1.86)	0.94 (0.40, 2.17)	0.94 (0.40, 2.17)	0.96 (0.83, 1.10)	1.05 (0.84, 1.31)	0.96 (0.89, 1.03)
NF-SAE									
No. studies	11	8	4	3	3	3	7	4	10
Sample size	2156	1923	1305	518	233	233	1578	578	1916
Effect estimate	1.18 (0.75, 1.86)	1.25 (0.77, 2.03)	1.41 (0.52, 3.81)	1.25 (0.50, 3.13)	0.71 (0.14, 3.55)	0.71 (0.14, 3.55)	1.28 (0.64, 2.57)	1.13 (0.47, 2.72)	1.14 (0.70, 1.85)

## Data Availability

Both publicly available datasets which can be found in original trial publications and additional information from study investigators were analyzed in this study. Some individual patient data are only available on request from the corresponding author with the permission of original study authors.

## Supplementary Materials

The following are available online at https://www.mdpi.com/article/10.3390/ph14121297/s1, Table S1: Risk of Bias across studies included in the safety meta-analysis.
