# Supplementary material for: Safety of Cerebrolysin for Neurorecovery after Acute Ischemic Stroke: A Systematic Review and Meta-Analysis of Twelve Randomized-Controlled Trials
Source: Pharmaceuticals (Basel). 2021 Dec 13;14(12):1297. doi: 10.3390/ph14121297 (PMC8708612; doi:10.3390/ph14121297)
Supplement: Supplementary file 1 [file pharmaceuticals-14-01297-s001.zip › pharmaceuticals-1486190-supplementary.pdf]

Table S1. Risk of Bias across studies included in the safety meta-analysis

Bias[1]<br>Study	Selection		Performance	Detection	Attrition	Reporting	Other
	**Appropriate generation of the allocation sequence**	**Conceal-ment of the allocation sequence**	**Blinding of participant and health care providers**	**Blinding of outcome assessors**	**Assessment of incomplete outcome data**	**Selective outcome reporting**	**Other biases**
Ladurner 2005 [21]	Low risk	Low risk[1]	Low risk	Low risk	Low risk[2]	Low risk[3]	Low risk[3]
Skvortsova 2004 [22]	Low risk[4]	Low risk[4]	Low risk[4]	Low risk[4]	Low risk[5]	Low risk[6]	Low risk[6]
Shamalov 2010 [23]	Low risk	Low risk	Low risk	Low risk	Low risk	Low risk	Low risk
Gharagozli 2017 [17]	Low risk	Unclear risk	Unclear risk	Unclear risk	Low risk[7]	Low risk	Low risk[7]
Heiss 2012 [18]	Low risk[8]	Low risk	Low risk	Low risk	Low risk[9]	Low risk[10]	Low risk[11]
Lang 2013 [19]	Low risk[12]	Low risk[12]	Low risk	Low risk	Low risk[13]	Low risk[14]	Low risk
Amiri-Nikpour 2014 [24]	Unclear risk	Unclear risk	Unclear risk	Unclear risk	Low risk	Low risk	Low risk
Muresanu 2016 [9]	Low risk	Low risk	Low risk	Low risk	Low risk	Low risk	Low risk
Guekht 2015 [20]	Low risk	Low risk	Low risk	Low risk	Low risk	Low risk	Low risk
Chang 2016 [25]	Low risk	Low risk	Low risk	Low risk	Low risk	Low risk	Low risk
Xue 2016 [26]	Low risk[15]	Low risk[15]	Unclear risk	Unclear risk	Low risk[16]	Low risk[16]	Low risk[16]
Stan 2017 [10]	Low risk	Low risk	Low risk	Low risk	Low risk	Low risk	Low risk
Risk of bias assessment for the safety evaluations was performed using all available data from original publications. In unclear cases, supplementary study documents were requested from the original authors (such as Study Protocols, Clinical Study Reports, etc.). Inclusion of any supplements is documented in the footnotes of this table.							

#1
In unclear cases, supplementary study documents were requested from the original authors (such as Study Protocols, Clinical Study Reports). Inclusion of any supplementary material into RoB assessments is documented in the footnotes of this table.

#2
The following information was available from original publication (J Neural Transm 2005), section Materials and Methods: *The investigators and all study personnel were blind to the random code assignment until the completion of the statistical analysis. For each patient a sealed envelope with information on the actual treatment dispensed was provided to the investigator for emergency cases. All envelopes remained sealed throughout the study.*

#3
The orginal publication (J Neural Transm 2005) provides the following information: *146 patients were randomised to two treatment groups and constituted the ITT population: 78 patients to the Cerebrolysin group and 68 patients to the placebo group. Of these patients, 67 of the Cerebrolysin group and 52 of the plaebo group completed the study. Reasons for the 25 cases of study continuation were death (6 Cerebrolysin, 6 placebo), serious adverse event (1 placebo), and consent withdrawn (3 Cerebrolysin; 9 placbeo).* Thus,13 cases of premature discontinuations were due to death or SAE, i.e. these cases are included in the present safety analysis and were not representing risk of bias. The remaining 12 cases represent 8% of the patients, which is well within the quality margin provided by the American Academy of Neurology (AAN) for class I studies (<20%). While imputation of missing values (LOCF) was reported for efficacy measures, this does no apply to adverse events or death, thus is not providing risk of bias for safety analyses.

#4
RoB assessment was based on information provided in the Clinical Study Report (CSR): *The allocation of patients to the treatment groups was done in a randomised fashion and both, the treating physician and the patient, were blinded with regard to this allocation. For each patient, a sealed emergency envelope was provided to the investigators. The emergency envelope contained information about the identity of the treatment administered. The investigators were instructed to open the envelopes only in case of immediate emergency that required the treatment code being immediately unblinded and the investigator could not call the*

PSO. In these cases the investigators had to provide a written explanation on the patient's CRF and inform the CSD and the PSO immediately.

#5
RoB assessment was based on information provided in the Clinical Study Report (CSR), reporting deaths on individual patient base in section 12.3.1.1 'Deaths', with detailed information on dose group (10mL / 50mL), cause of death and study day. Adverse events were reported per MEDRA System Organ Class as well as per MEDRA Low Level Term. Serious adverse events were additionally reported on individual patient base.

#6
RoB assessment was based on information provided in the Clinical Study Report (CSR) and in the study protocol. According ot he supplementary material the study was conducted *with the permission of the Committee on Ethical Issues of the Russian State Medical University (RSMU). Sponsor: EWEBE Pharma Ges.m.b.H Nfg. KG, Austria*.

#7
RoB assessment was based on information provided in the Clinical Study Report (CSR). Out of the 23 patients with premature discontinuation, four patients withdrew due to adverse events, and 3 patients died. These patients are included in the safety analysis and are not representing risk of bias. The remaining 16 patients with premature discontinuation are 'lost to follow-up', however lost to follow-up was <u>after</u> discharge in all these cases.

#8
RoB assessment was based on information provided in the Clinical Study Report (CSR): *Random permuted block design was used for randomisation of patients. The randomisation schedule was drawn up by EBEWE being independent from all other study related procedures and was kept under closure by the JSW Data Management group exclusively. All other members of the clinical project team, all investigators and all patients entered into the study were blind to treatment allocation*.

#9
RoB assessment was based on information provided in the Clinical Study Report (CSR). The CSR reported 10.9% premature discontinuations (13/119). This is well in the range of the class I definition of the AAN (<20%). The single reasons for premature discontinuation were reported in the CSR, separately per treatment group. The majority of the premature discontinuations were due to adverse events or deaths, i.e., these patients were included in the safety analysis, not representing risk of bias. While imputation of missing values (LOCF) was reported for efficacy measures, this does no apply to adverse events or death, thus not providing risk of bias for safety analyses.

#10
RoB assessment was based on information provided in the Clinical Study Report (CSR). Deaths and severe adverse event were reported with patient narratives in section 12.3.2 of the Clinical Study Report including relevant date information for safety evaluation.

#11
RoB assessment was based on information provided in the Clinical Study Report (CSR). This study had an *a priori* planned sequential design including formal sample size calculation for the sequential design: *At each interim analysis the modified Rankin Scale scores will be analysed using a "proportional odds" model with stopping rules defined using a boundaries approach known as the Triangular test. Statistics Z (a measure of the overall treatment difference) and V (a measure of the amount of information available) will be computed and plotted graphically. Depending on the position of the interim analysis point on the plot relative to the pre-defined stopping boundaries, a decision will be made to continue or to stop the study.*

#12
RoB assessment was based on information provided in the Clinical Study Report (CSR) andf in the Study Protocol: *The study specific randomization code was prepared using the validated program RANCODE in a validated working environment. A variable length, blocked randomization scheme was used, in which treatment assignment in a ratio 1:1 was stratified by clinical centre. The size of the blocks was intentionally not given in this protocol so that the clinical site was unaware of this feature. The double-blind study medication labels identified only the unique randomization number, which was the same as the patient number. All investigators and study personnel were blinded as to the treatment allocated to a specific random number.*

#13
RoB assessment was based on information provided in the Clinical Study Report (CSR). The Clinical Study Report reports 16.8% premature discontinuations (Cerebrolysin 14.6%, placebo 18.9%) with specification of the reasons per treatment group. This is well in the range of the class I definition of the AAN (<20%). The rate of lost to follow-up was very low: 1.9% (Cerebrolysin 1.9%, placebo 1.9%). Overall, risk of bias due to incomplete safety data was assessed as „low".

#14
RoB assessment was based on information provided in the Clinical Study Report (CSR). In the CSR, causes of death were provided on individual patient base per treatment group. The same applies to the nature of serious adverse events, allowing in-depth re-assessments. Overall, risk of bias due to selective reporting of safety outcomes was assessed as „low".

#15

The original publication Xue et al. 2016 provides the following information: *Randomization was performed by means of computer-generated numbers through software by a third party who was not involved in patient management. The random numbers were placed in concealed envelopes.* Following these statements the placement of random numbers in concealed envelopes was presumably performed by the same third party which generated the random sequence (which is the common procedure). Thus, associated risk of bias was assessed as "low".

#16

This was a 3-group trial, including DL-3-n-butylphthalide. RoB assessment for the present safety meta-analysis was performed using the data from the the two arms of interest (Cerebrolysin and placebo). Information from ClinicalTrials.gov was used as supplementary information source for evaluation of premature discontinuations: Out of a total of 57 enrolled patients (28 Cerebrolysin and 29 placebo patients), 17 premature discontinuations were reported. Out of these 15 patients, 9 patients were "lost to follow-up". Upon special request, detailed information from the authors on the nature of these cases could be obtained per treatment group: 2 vs. 2 premature discontinuations due to investigator decision: in all 4 cases due to surgical intervention related to stroke; 2 vs. 2 withdrawals by subject: no known safety reasons; 4 vs. 5 lost to follow-up: no information on safety background. While imputation of missing values (LOCF) was reported for efficacy measures, this does no apply to adverse events or death, thus is not providing risk of bias for safety analyses. The study was investigator-intiated and sponsored by the Hospital affiliated to the Shanghai Jiao Tong University School of Medicine (additional information on sponsors and investigators could be retrieved from ClinicalTrials.gov: *'Sponsor and Collaborators: Shanghai 6th People's Hospital; Investigators: Study director Lixia Xue, M.D., Ph.D., Shanghai 6th People's Hospital).*
